# Outdoor oil palm fruit ripeness dataset

**DOI:** 10.1016/j.dib.2024.110667

**Published:** 2024-06-26

**Authors:** Zaid Omar, Anwar P.P. Abdul Majeed, Munirah Rosbi, Shuwaibatul Aslamiah Ghazalli, Hazlina Selamat

**Affiliations:** a Faculty of Electrical Engineering, Universiti Teknologi Malaysia, 81310 UTM Johor Bahru, Malaysia; b School of Engineering and Technology, Sunway University, 47500 Selangor, Malaysia

**Keywords:** Fresh fruit bunchp, Image-based classification, Fruit maturity, Natural environment

## Abstract

This dataset comprises oil palm fresh fruit bunch (FFB) images that may potentially be used in the study related to fruit ripeness detection via image processing. The FFB dataset was collected from palm oil plantations in Johor, Negeri Sembilan, and Perak, Malaysia. The data collection involved acquiring pictures of FFB from various angles and classifying them based on their ripeness level, categorised into five classes: damaged bunch, empty bunch, unripe, ripe, and overripe. An experienced grader carefully labelled each FFB image with the corresponding ground truth information. The dataset provides valuable insights into the colour variations of FFBs throughout their ripening process, which is essential for assessing oil quality. It includes observations on the external fruit colours as well as characteristics related to the presence of empty sockets in the FFB as a key indicator of ripeness. The reusability potential of this dataset is significant for researchers in the field of oil palm fruit classification and grading, which requires an extensive outdoor dataset that comprise FFB's both on the tree and on the ground. Our work enables the development and validation of machine learning pipelines for outdoor automated FFB grading. Furthermore, the dataset may also support studies to improve oil palm cultivation practices, enhance yield, and optimise oil quality.

Specifications Table

**Table:** 

Subject	Computer vision and pattern recognition
Specific subject area	Computer vision and pattern recognition, artificial intelligence, computer science applications, deep learning, fruit ripeness recognition.
Data format	Labelled raw image data in .jpg
Type of data	Raw figures
Data collection	FFB images were collected using a smartphone under natural lighting from various angles in a palm oil plantation. Each image was labelled based on five ripeness levels.
Data source location	All of the images were obtained from palm oil plantations in Malaysia. Some were collected from a plantation in Perak, and others were from Batu Pahat, Johor.
Data accessibility	Repository name: Zenodo [1] Direct URL to data: https://doi.org/10.5281/zenodo.11114885

## 1 Value of the Data

• These data are valuable because they present the real-world scenario of FFBs in outdoor conditions. The data show various FFBs still attached to the tree (pre-harvesting) as well as on the ground (post-harvesting).
• These data can be used to classify oil palm FFB ripeness.
• The dataset is also invaluable for testing color correction algorithms to standardise colour perception, addressing image variance due to diverse natural lighting conditions outdoors. For instance, outdoor images often have a warm temperature, making unripe bunches appear ripe. Colour correction could effectively adjust color perception, improving the accuracy of the classification system.

## 2 Background

The oil palm fruit, scientifically termed Elaeis guineensis, originated in West Africa and spread to Southeast Asian countries like Malaysia, Indonesia, and Thailand [2]. A Tenera species fruit typically ripens in 20 to 22 weeks, exhibiting colour changes from black to reddish, then to orange [3]. Ripeness, crucial for oil quality, is determined by observing skin colour [4]. The Malaysian Palm Oil Board (MPOB) guidelines classify FFB ripeness into four categories: unripe, underripe, ripe, and overripe. Purplish-black indicates unripe-ness, darkish-red signifies overripe-ness, while reddish-orange and reddish-purple denote ripe and underripe, respectively [5]. Additionally, MPOB considers FFBs with 1% to 9% empty sockets due to loose fruits as underripe, while ripe FFBs have 10% to 50% empty sockets [6].

## 3 Data Description

The acquired outdoor FFB dataset comprises 466 FFB images with five classes (damaged, empty, overripe, ripe and unripe). According to the MPOB [7], there are 14 classes of FFB bunches, including four ripeness categories and several quality indicators. In this particular data collection, three classes of fruit ripeness and two classes of fruit quality were identified, resulting in five classes of FFB included in the dataset. While some studies identify four ripeness classes for FFB classification [8], there is often overlap between the unripe and underripe categories. This overlap does not significantly affect FFB classification, as the primary goal is to identify and harvest ripe bunches while leaving unripe or underripe bunches on the tree.

**Fig. 1:**
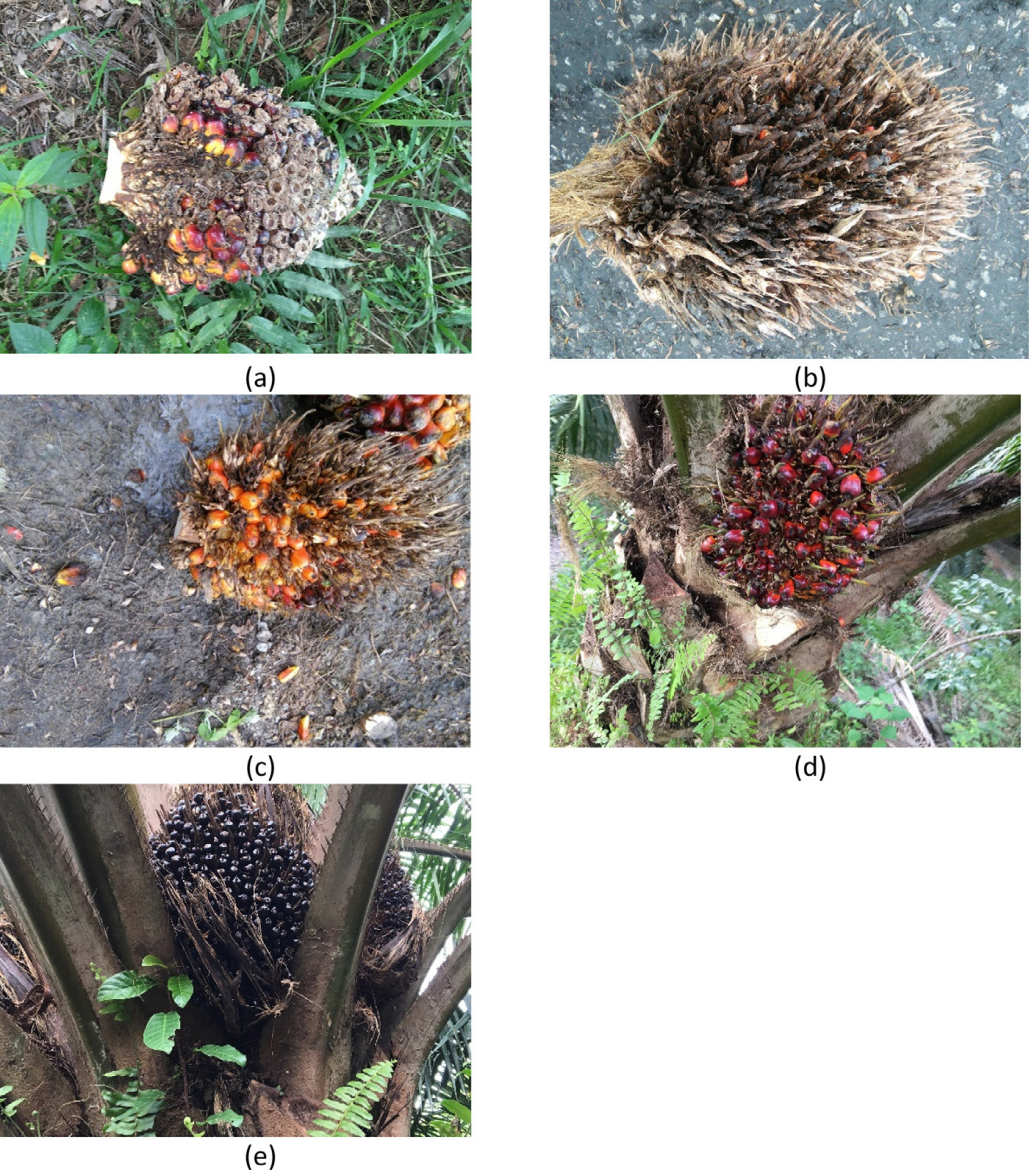
Examples of the FFB image dataset (a) Damaged, (b) Empty bunch, (c) Overripe, (d) Ripe, (e) Unripe [1].

Both on-tree and on-the-ground images were included in the dataset. The image size varies, ranging from 757×568 pixels to 3024×4032 pixels. The images were separated into 371 training images in a file named *FFBtrain* and 95 test images in *FFBtest*. The image labelling follows the format of the file's name as described in Table 1. Table 2 illustrates the allocation of images across different FFB classes, along with the corresponding percentages of test data for each class. Fig. 1 shows example images of the dataset.

**Table 1:** Name format of the image files.

FFB Classes	Example of file's name	
	Training image	Test image
Damaged bunch	*‘TrainDamaged(1).jpg’*	*‘TestDamaged(1). jpg’*
Empty bunch	*‘TrainEmpty(1). jpg’*	*‘TestEmpty(1). jpg’*
Overripe	*‘TrainOverripe(1). jpg’*	*‘TestOverripe(1). jpg’*
Ripe	*‘TrainRipe(1). jpg’*	*‘TestRipe(1). jpg’*
Unripe	*‘TrainUnripe(1). jpg’*	*‘TestUnripe(1). jpg’*

**Table 2:** Dataset distribution.

FFB Classes	Train	Test	Total for each class	Percentage of test data
Damaged bunch	12	3	15	20.0%
Empty bunch	9	3	12	25.0%
Overripe	60	14	74	18.9%
Ripe	160	41	201	20.4%
Unripe	130	34	164	20.7%
Total data	371	95	466	20.3%

## 4 Experimental Design, Materials and Methods

**Fig. 2:**
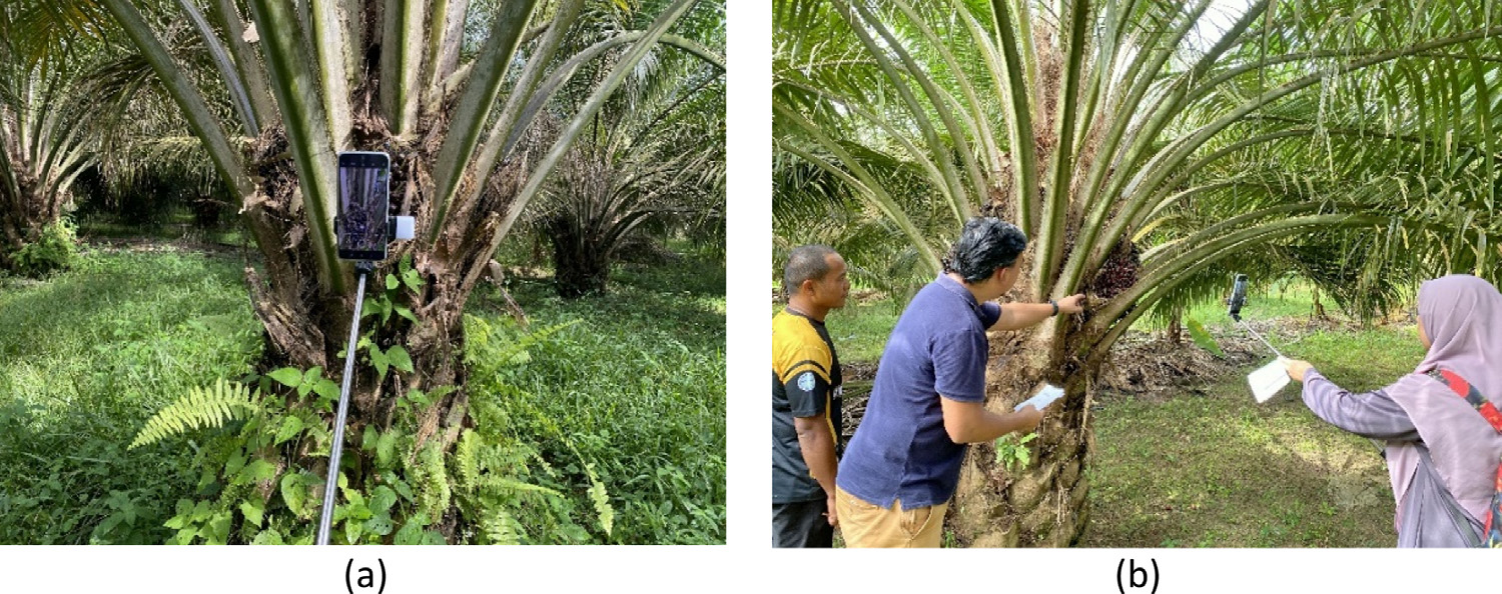
(a) Smartphone and monopod used in data acquisition, (b) Images taken from different angles [9].

Data acquisition was aimed at capturing a diverse array of FFB images under natural lighting conditions. The goal was to develop an algorithm that would demonstrate robustness and accuracy across various weather conditions and lighting scenarios. A portion of the images was sourced from the Malaysian Palm Oil Board (MPOB) in Negeri Sembilan, some were obtained from a palm oil plantation situated in Johor, and others were from Perak, Malaysia. Capturing FFB images involved affixing a smartphone to a monopod, ensuring images were captured from various angles (refer to Fig. 2). Both the iPhone 11 and Xiaomi 12 Lite were used for image collection. Automatic camera settings were applied to capture random RGB images, ensuring variability for effective image classification. A total of 176 fruit samples were used, with an average of three to four images captured from different angles for each bunch. The distribution of the FFB samples is detailed in Table 3.

**Table 3:** Number of FFB samples for each class.

Classes	Number of fruit samples (training image)	Number of fruit samples (test image)
Damaged	5	3
Empty	6	2
Ripe	68	10
Unripe	48	7
Overripe	24	3

Prior to image acquisition, an experienced grader has been assigned to classify the FFBs according to their ripeness levels. Subsequently, each image underwent a labelling process, denoting it with the relevant ground truth information to specify whether the fruit was damaged, empty, unripe, ripe, or overripe. Analysis of this dataset aims to uncover the variations inherent in outdoor FFB images, thereby assisting in defining the research problem.

The dataset underwent testing on the YOLOv4-Tiny deep neural network platform, with a custom data augmentation technique employed to enhance dataset diversity. Five distinct data augmentation methods (refer to Table 4) were utilised, leading to a six-fold increase in the dataset size. The initial set of 371 training images was utilised to train the YOLOv4-Tiny for direct deep learning classification, resulting in a test accuracy of 82.11%. Furthermore, the system was compared with CNN-based deep learning, with the specific detailsprovided in Table 5, yielding an accuracy of 55.79%.

**Table 4:** Data augmentation techniques applied to the dataset [9].

Image augmentation method	Augmented image example
Altering colour in HSV colour space	
Image translation at the x-axis	
Random contrast adjustment	
Overlay rain mask	
Overlay shadow mask

**Table 5:** Hyperparameter for CNN classifier.

Training images	371
Hardware resource	Single CPU
Epoch	100
Learning rate	0.01
Validation frequency	30 iterations

## Limitations

The dataset is biased due to its limited representation of on-tree data, particularly regarding damaged, empty, and overripe bunches resulting from detachment by harvesters or falling from the tree. This imbalance in observation leads to a disproportionate number of ripe and unripe fruits on the tree within the dataset. Additionally, the dataset exhibits non-standardised class distributions, with significant variations, especially in the number of empty and damaged bunches. This disparity arises from the significantly lower total population of empty and damaged bunches compared to other classes in a maintained oil palm plantation.

Furthermore, the data collection procedure using a monopod could not reach oil palm trees taller than 3 metres. Therefore, future upgrades involving drones or other mechanisms for capturing images are necessary. This data collection utilised only two types of smartphones, the Xiaomi 12 Lite and iPhone 11, resulting in a limited diversity of datasets across devices. Therefore, it would be beneficial to use a wider range of smartphone models to enhance the diversity of images captured.

## Ethics Statement

Our study does not involve experiments with animals or humans. Therefore, we confirm that our research strictly adheres to the guidelines for authors provided by Data in Brief regarding ethical considerations.

## CRediT authorship contribution statement

**Zaid Omar:** Conceptualization, Supervision, Project administration, Writing – review & editing. **Anwar P.P. Abdul Majeed:** Visualization, Writing – review & editing. **Munirah Rosbi:** Methodology, Formal analysis, Investigation, Writing – review & editing. **Shuwaibatul Aslamiah Ghazalli:** Formal analysis, Investigation. **Hazlina Selamat:** Conceptualization.

## Declaration of Competing Interest

The authors declare that they have no known competing financial interests or personal relationships that could have appeared to influence the work reported in this paper.

## Data Availability

Outdoor Tenera Oil Palm Fruit Image: FFB Dataset (Original data) (Zenodo)
